# The nephrology eHealth-system of the metropolitan region of Hannover for digitalization of care, establishment of decision support systems and analysis of health care quality

**DOI:** 10.1186/s12911-019-0902-0

**Published:** 2019-09-02

**Authors:** L. Pape, N. Schneider, T. Schleef, U. Junius-Walker, H. Haller, R. Brunkhorst, N. Hellrung, H. U. Prokosch, B. Haarbrandt, M. Marschollek, M. Schiffer

**Affiliations:** 1 Department of Pediatric Kidney, Liver and Metabolic Diseases, Hannover Medical School, Hannover, Germany; 2 Institute for General Practice, Hannover Medical School, Hannover, Germany; 3 Department of Nephrology and Hypertension, Hannover Medical School, Hannover, Germany; 4 Department of Nephrology, Angiology and Rheumatology, KRH Regional Hospital Hannover Siloah, Hannover, Germany; 5 Symeda GmbH, Braunschweig, Germany; 6 Department of Medical Informatics, Biometrics and Epidemiology, Chair for Medical Informatics, Friedrich-Alexander-University Erlangen-Nürnberg (FAU), Erlangen, Germany; 7 Peter L. Reichertz Institute for Medical Informatics University of Braunschweig - Institute of Technology and Hannover Medical School, Hannover, Germany; 8 Department of Nephrology, University Hospital Erlangen, Erlangen, Germany

**Keywords:** Kidney transplantation, Interoperability, Graft survival, Patient survival, eHealth, Quality of life, Clinical decision support system

## Abstract

**Background:**

Even though a high demand for sector spanning communication exists, so far no eHealth platform for nephrology is established within Germany. This leads to insufficient communication between medical providers and therefore suboptimal nephrologic care. In addition, Clinical Decision Support Systems have not been used in Nephrology until now.

**Methods:**

The aim of NEPHRO-DIGITAL is to create a eHealth platform in the Hannover region that facilitates integrated, cross-sectoral data exchange and includes teleconsultation between outpatient nephrology, primary care, pediatricians and nephrology clinics to reduce communication deficits and prevent data loss, and to enable the creation and implementation of an interoperable clinical decision support system. This system will be based on input data from multiple sources for early identification of patients with cardiovascular comorbidity and progression of renal insufficiency. Especially patients will be able to enter and access their own data. A transfer to a second nephrology center (metropolitan region of Erlangen-Nuremburg) is included in the study to prove feasibility and scalability of the approach.

**Discussion:**

A decision support system should lead to earlier therapeutic interventions and thereby improve the prognosis of patients as well as their treatment satisfaction and quality of life. The system will be integrated in the data integration centres of two large German university medicine consortia (HiGHmed (highmed.org) and MIRACUM (miracum.org)).

**Trial registration:**

ISRCTN16755335 (09.07.2019).

## Background

The digitization of medicine will fundamentally change access and links to health and disease data over the next few years. Nephrology patients are a particular interesting subpopulation as a use case with a large body of medical data since both children and adults with chronic renal insufficiency suffer from secondary (or primary) diseases of other organ systems. In addition, in the large population of patients undergoing dialysis therapy, there is considerable generation of data from renal replacement therapy or transplantation and its follow-up, as well as from the complex medication requirements.

In Germany, patients with chronic kidney disease are generally treated on an outpatient basis by (pediatric) nephrologists in private practices or in other outpatient care institutions, e.g. the Kuratorium für Dialyse und Nierentransplantation (KfH), the Patientenheimversorgung (PHV), or Davita, Nephrocare, BBraun and others. In addition, many patients have comorbidities or intercurrent conditions, treated by general practitioners / ambulant pediatricians. However, records are typically kept in separated IT systems. Various software systems exist in the outpatient GP setting, but in the nephrological context data documentation is usually (> 90%) via the GP information system Nephro7. Nephrology patients often require inpatient care (e.g. due to dialysis [access] problems, infections, transplants, transplant complications, other comorbidities). This is usually provided in nephrology departments at Hannover Medical School (MHH) (Hannover Metropolitan Region) and Klinikum Siloah (Klinikum Region Hannover). Here, in the two Hannover-based hospitals, data are documented within the hospital information systems using SAP-ISH-med using the electronic medical record ISH-med by Cerner. In practice today, patient information between the different sectors of the health system are only exchanged through paper reports or phone calls. This leads to information and data losses and thus to a quality deterioration in the medical treatment of (pediatric) nephrological patients. The intelligent support of patient care within a multi-stakeholder environment through decision support systems, as well as quality management and comparison between different regions, is therefore currently not feasible.

In other countries, it has already been demonstrated that digitization of nephrology data can lead to better patient care [1–3]. Apart from our regional kidney transplant project KTX 360° [4], funded by the Innovation fund of the Joint Committee (G-BA) of the Federal Republic of Germany (www.ntx360grad.de), no eHealth networks in nephrology have yet been established in Lower Saxony or Germany as a whole. Within this project, initiated in 2017, several steps have been made towards eHealth systems for the subpopulation of patients with kidney transplants. In cooperation with Symeda GmbH, a small Braunschweig-based company, a cross-sectoral communication platform for kidney transplants was established, serving more than 50 participating (pediatric) nephrologists in Lower Saxony and the KTX Center Hannover Medical School. This platform is additionally used for the exchange of structured medication plans, documents in PDF format, and individual laboratory values. It further enables teleconsultations [4]. The first steps towards interfacing with the Nephrology GP system Nephro7 have also been established. An interface with a hospital’s electronic medical record system has, however, not yet been established [5]. General practitioners and pediatricians in Germany use a large variety of different commercial software systems, so that implementing interfaces to all such systems would not be reasonable. For them, the most important functions would be login access to medical data of patients treated by them and a chat function for teleconsulting.

Within the German Federal Ministry of Education and Research (Bundesministerium für Bildung und Forschung [BMBF])-funded HiGHmed consortium [6], a new approach to enable data sharing and intelligent computing of electronic health records and research data collections is currently being implemented through components of data integration centres established at eight German university hospitals [7]. The long-term vision for HiGHmed is to develop a joint technology platform across all university hospitals and research units that will assist and promote data-based knowledge-building and decision-making processes in patient-centered diagnostics, treatment and care. This includes profiling these standards and agreeing on common semantics for real-life implementation. Data that is integrated using the HiGHmed Platform shall strictly fulfil the FAIR principles of data sharing [8]. This means, that the envisioned architecture makes all relevant data findable, accessible, interoperable and reusable if the patient has given his or her consent.

Through consequent use of open system architectures, open data models and open application programming interfaces (e.g. Integrating the Healthcare Enterprise [IHE], openEHR and Fast Healthcare Interoperability Resources [FHIR]), the “FAIR”, vendor neutral and extensible health data platform will provide the means to enable and promote research collaboration and re-use of data collections and allow the seamless sharing of data between clinical applications and research databases. This open approach shall prevents the data from becoming locked in proprietary databases. Thereby the data within a patient’s electronic health record can finally outlast ephemeral software products whose lifecycle (10–15 years) is normally significantly shorter than the average life expectancy of a human.

Through the development and use of open, standardized and computable domain models, data becomes accessible to algorithmic processing, analytics and process management. This is the prerequisite for any intelligent reuse of the data. Based on the main platform components of HiGHmed, the first projects using artificial intelligence and machine learning have been conducted at MHH, i.e. a decision-making system in pediatric intensive care medicine was developed and the application of openEHR archetypes helped to bridge the interoperability gap between local infrastructures and a decision support system in a prototype implementation [9].

Using the same technical and semantic foundation, the incorporation of a clinical decision support tool within a regional health network in Sweden has shown significant improvements regarding the adherence to guidelines on anticoagulant therapy in patients with atrial fibrillation at risk of stroke [10].

The reference architecture will be scalable and open for adoption by additional partner hospitals, as well as for many solution developers, to provide innovative applications, e.g. for data integration or analytics. Therefore, HiGHmed provides an platform ecosystem for integrating electronic medical record systems, GP office software and the KTX 360° health information exchange platform to be used for all regional nephrology patients [7, 8, 11, 12]. MIRACUM [13] is a second large consortium (comprising ten German university hospitals) funded in the German medical informatics initiative [14]. The university hospital in Erlangen, is coordinating the MIRACUM project and is therefore the ideal partner in our project with the goal to create an interoperable IT-solution that is feasible for most university hospitals in Germany.

The underlying platform of NEPRHO-DIGITAL will further provide patient access to important data and thus lead to a significant improvement in the treatment of (pediatric) nephrological patients by providing easy retrieval of important data or teleconsulting for all physicians involved. From a future perspective, this system could be transferred to other MHH-based specialist treatment settings, establishing constant data sharing with ambulant care providers, optimizing quality of care.

Due to the distribution of data in different IT systems, there has been no machine learning of prediction models from routine nephrologic data until now and it has not been possible to compare treatment quality between different regions.

## Methods/design

**Fig. 1:**
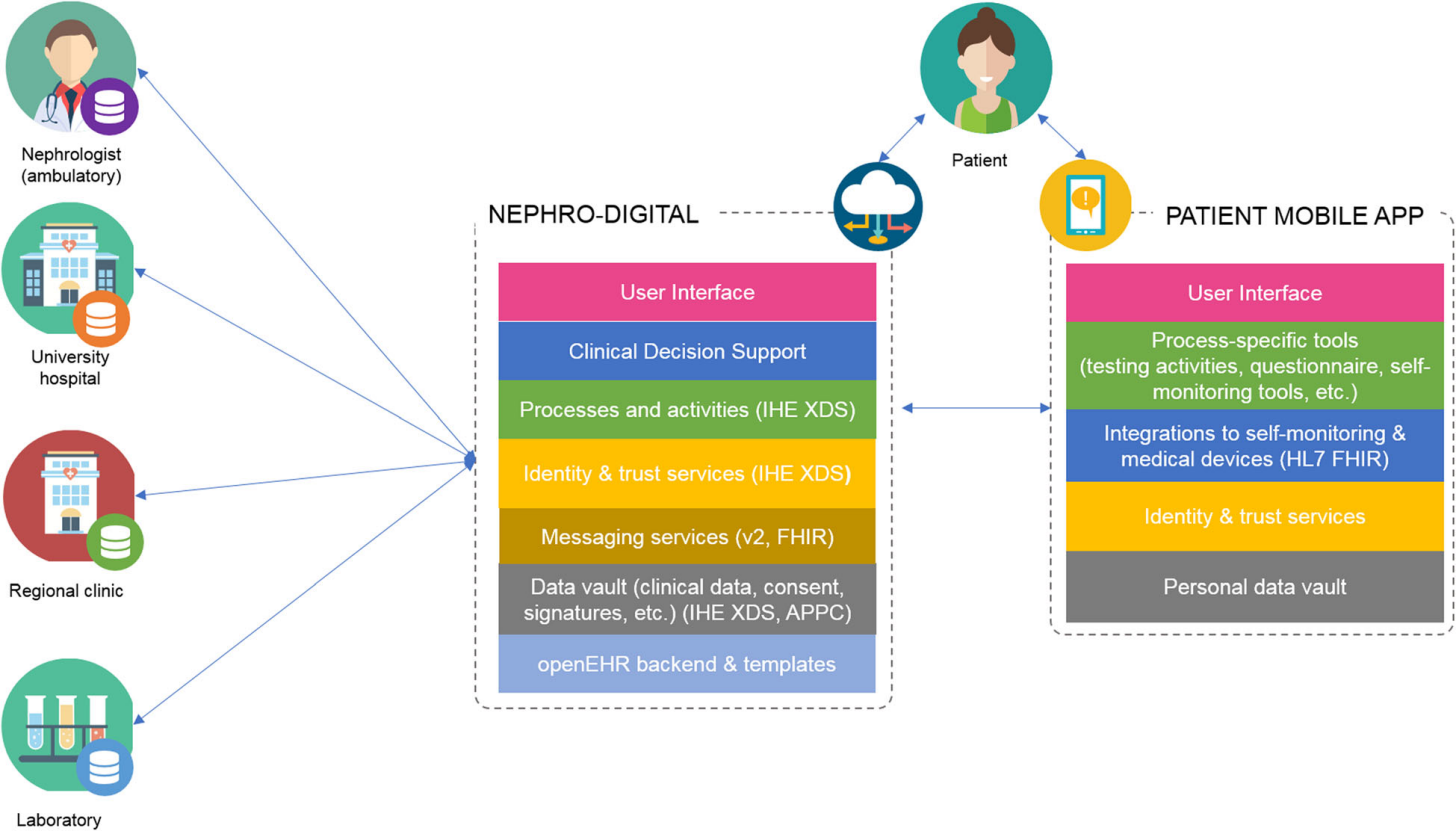
Structure of NephroDIGITAL

Within the framework of the project NEPHRO-DIGITAL the functions of the existing KTX360° health information exchange platform, are to be extended for non-kidney transplanted nephrology patients to support the care of all types of (pediatric) nephrology patients. These include automatic comparison of dialysis treatment data, the transfer of laboratory data, diagnoses, and comorbidities, as well as other patient information. An automatic exchange of the so called ‘German federal consistent’ medication plan and all laboratory values between physicians should be achieved using this enhanced Health platform. It is our goal that in its final deployment stage the platform shall fully replace paper reports exchanged between doctors and also include data that is generated by the patients themselves. For acquiring a second opinion, teleconsultations between the service providers in the different health care sectors can take place at any time via the system. We aim to develop and evaluate an open standards-based approach for establishing a CDSS upon this platform with an exemplary system for automated prognosis of kidney function and the development of cardiovascular disease in nephrology patients (Fig. 1). This will permit retrieval of dynamic facts in a standardized and unambiguous form to facilitate clinical decision support in this complex area with the goal of detecting patients with worsening renal function or cardiovascular comorbidity early enough for therapeutic intervention. We further aim to compare treatment data between the Hannover and Erlangen/Nürnberg regions in order to show possible differences in treatment quality and thus identify how nephrological care can be improved. At its end this will also serve to assess the transferability of our technical approach.

### Hypothesis

The primary hypothesis underlying the project is that the digitization of nephrology data and processes presented above will result in a significant improvement in patient care and, in addition, will lead to cost savings in the health care system by reducing hospitalization and redundant diagnostics (hospital, nephrologist, general practitioner/pediatrician) and treatment and by improving quality of life.

### Working plan

The planned digitization of nephrology can only be achieved in an interdisciplinary team. The medical basis for the necessary structure of the eHealth platform is derived from (pediatric) nephrology whereby (pediatric) nephrologists, pediatricians and general practitioners, together with patients, will be the primary users of the new digital nephrology. The evaluation of the use of the new regional nephrological digital network will be carried out by a health scientist.

The CDSS to be developed can serve as a blueprint for future artificial intelligence systems that can be implemented in other health networks in order to improve care. As the applications built as part of this project use the HiGHmed platform infrastructure based on open interoperability standards that can interface that of the German Telematik infrastructure, our system will also be ready for use in different regions. With the inclusion of interfaces to the most commonly used IT system within German hospitals (MHH and Klinikum Region Hannover: SAP-ISH-med) our eHealth platform will be ready for use in the majority of hospitals. Making the eHealth platform also interoperable with the MIRACUM data integration centre concept implemented at Erlangen University (and at nine other German university hospitals) will ensure that our eHealth platform will be compatible with the architecture of most German university hospitals.

#### General practice: assessment of needs, acceptance, feasibility in GP/pediatric care

Relevant information and exchange tasks for primary care providers (GPs, pediatricians) will be identified. Feasibility aspects will be assessed multiperspectively and longitudinally using mixed methods of health service research (qualitative/quantitative) to elucidate barriers and facilitators for acceptance and use. Subsequently, the eHealth platform shall be adapted to identified stakeholder needs in order to warrant its use in the ambulant care sector. Further, this may act as a use case for technical interfaces to improve cross-sectoral cooperation with primary, secondary and tertiary care in other specialties.

Objectives:

- To identify data, information and exchange needs of primary care providers
- To assess barriers and facilitators for eHealth platforms use in primary care providers

Qualitative Methods: Interviews with key stakeholders (GPs, Hausärzteverband) (t0, t1), survey, final workshop/focus groups.

#### Nephrology

Nephrologic parameters for the eHealth platform will be defined and standardized between the caregivers participating in the project. The implementation of the eHealth platform into routine care will be organized and adapted to the needs arising within the project. A main task for the physician executing SP2 will be the constant communication with the caregivers within both tasks with the goal of achieving high acceptance of the eHealth platform. In addition, the parameters for the the patients´ data provision into the eHealth platform will be developed together with the patient organizations in order to create a tool that will be widely used for nephrology patients. The nephrologist will also be responsible for the medical aspects of developing and implementing the CDSS.

#### Semantic interoperability, data integration and predictive modeling

The semantic information models describing the data items required within the domain of nephrology will be defined. These models will serve as defined semantic models that can be used for multiple purposes, including the definition of data capture forms in electronic health records communication interfaces, database schemas, data validation algorithms, data querying, etc. The creation of the domain models will be closely aligned to the modelling activities in HiGHmed, the openEHR Foundation and related SDOs (standards developing organizations) such as HL7 and IHE. Based on the representation of the data within the open platform, machine learning and data analytic algorithms will be developed that allow distributed computing of the data, permitting privacy preserving use of data from the deployments of the Hannover and Erlangen platforms.

#### Advancement of the eHealth platform

The goal of this subproject is to further develop the eHealth platform with regards to the needs of patient care in the nephrology setting. The architecture will be based on the findings of the HiGHmed project and therefore builds up the base for semantic interoperability. This is the prerequisite for big data analytics in health care. By 2021 all German health insurance companies will be obliged to offer apersonal electronic health record to their customers. This will provide the perfect infrastructure for NEPHROLOGIE-DIGITAL (NEPHRO-DIGITAL), because the eHealth platform will offer basic services, such as authentication, security and medication history. On the other hand, these systems will not implement the needs for specialized medical fields, such as nephrology. Symeda will ensure that NEPHRO-DIGITAL will be compliant with the German telematics innfrastructure requirements.

#### Interface and establishment of NEPHRO-DIGITAL in the Erlangen / MIRACUM network

To demonstrate transferability of the concept the regional networks in southern Germany with the University Hospital of Erlangen (UKEr) will be used. Erlangen is participating in patient recruitment within the KTx360° program and thus uses the identical eHealth platform and an adapted network with private practices and regional dialysis providers. Moreover, UKEr is part of the German medical informatics MIRACUM consortium. One of the aims of MIRACUM is to establish data integration centers that will be embedded in the hospital IT infrastructure and can facilitate the collection and exchange of data within the consortia university hospitals. Thusm NEPHRO-DIGITAL could emerge as a common use case between MIRACUM and HiGHmed to illustrate interoperability between both BMBF-funded initiatives, creating a first state- and sector-spanning care and research platform in Germany.

##### Evaluation

Evaluation is planned and carried out based on S.M.A.R.T. (specific, measurable, attainable, realistic and timely) criteria. Here, health data of the patients (from the eHealth platform, as well as from the nephrology GP system and the hospitals EMR) in the project, as well as of historical control patients, must be extracted, exported and analyzed in cooperation with MHH biometry resources.

S.M.A.R.T. criteria:

**A** Evaluation of care provider needs on data and information sharing, acceptance and feasibility (qualitatively, interviews with GPs / pediatricians) at 30 months.

**B** Implementation of a cross-sector eHealth platform including teleconsultations and patient access. Indicator: 50% of patients with shared use of the platform after 30 months.

**C** Implementation of a decision support system for GFR deterioration and cardiovascular comorbidities. Indicator: System is implemented at 30 months.

**D** Comparison of intersectoral treatment quality between Hannover Region and Erlangen. Indicator: Analysis performed at 30 months.

**E** Reduction of complications of nephrological diseases. Indicator: Reduction of complications requiring inpatient treatment. Indicator: A 10% reduction of hospital expenditure within 30 months compared to a control cohort (historical control patients who did not participate in the program).

**F** Reduction in cardiovascular events. Indicator: The number of hospitalizations due to cardiovascular events will be reduced > 10% within 30 months.**G** After the establishment of NEPHRO-DIGITAL, participant quality of life will improve significantly. Indicator: In the mental subscale of the SF-12, 80% of the participating patients will reach values which do not differ from population norm data within 30 months.

**H** Is the use of the new digital elements and the quality of life gender-dependent? Does a migration background play a role in the acceptance of digital medicine? Indicator: Participation rate in subgroups is not significantly different after 30 months.

## Discussion

The Nephro Digital project contributes to digitalization of nephrology with the aim of achieving a substantial improvement in the care of nephrologic patients. The focus of the project is the development of a new interoperable platform for health professionals and nephrologic patients. In an interdisciplinary consortium of clinical centers, computer scientists and software experts as well as an innovative e-health SME, a CDSS for nephrologic patiens will be developed. This utilizes data (e.g., lab values, weight, and blood pressure) from different sources to detect early worsening of health and to prognose future deterioration of kidney function and cardiovascular events. The CDSS ​​system will inform the physician about these risks. The physician can then structurally integrate these parameters, which have so far not been systematically collected, into his decision-making and thus has a better data basis for the diagnosis and therapy decision. Thus, medical experience and CDSS complement each other in the sense of an “augmented intelligence” from which both doctor and patient benefit.

If the positive effects of our project could be confirmed and if the transferability in other regions is confirmed by the implementation of the CDSS in Erlangen, it is planend to implement the structures into routine care in the region Hannover and in other areas that are interested.

## Data Availability

Not applicable.

## Abbreviations

CDSS: Clinical Decision Support System
eFA: Electronic case file
GP: General Practitioner
IHE: Integratig the Healtcare Enterprise
KRH: Regional Hospital Hannover
KTX: Kidney transplantation
MHH: Hannover Medical School
SDO: Starrd developing organisations
UKER: University Hospital of Erlangen
